# Antibacterial Activity of a Mouthwash Containing *Plectranthus amboinicus* (Lour.) Spreng Extract and *Lippia sidoides* Cham. Essential Oil

**DOI:** 10.1002/cbdv.202503664

**Published:** 2026-03-20

**Authors:** Wilma Francisca da Silva, Carla de Fatima Alves Nonato, Débora Odília Duarte Leite, Fabiola Fernandes Galvão Rodrigues, Fazia Fernandes Galvão Rodrigues, Ana Cecília Calixto Donelardy, José Galberto Martins da Costa

**Affiliations:** ^1^ Postgraduate Program in Health Education Dr. Leão Sampaio University Center, Av. Maria Letícia Leite Pereira, Campus Lagoa Seca, 63040‐405 Juazeiro do Norte Ceará Brazil; ^2^ Natural Products Research Laboratory Universidade Regional do Cariri, Rua Coronel Antônio Luíz, 1161, Pimenta, 63105‐010 Crato Ceará Brazil

**Keywords:** antibacterial activity, flavonoids, mouthwash, terpenes

## Abstract

This study investigated the development of a mouthwash formulated with Plectranthus amboinicus leaf extract (PaEE) and Lippia sidoides leaf essential oil (LsOE), exploring their antibacterial potential. Antimicrobial activity was evaluated in vitro using the broth microdilution method to determine the minimum inhibitory concentration (MIC) against Streptococcus mutans and Staphylococcus aureus. Chemical analysis of LsOE by GC/MS identified active compounds such as thymol and carvacrol, while PaEE demonstrated the presence of flavonoids, anthocyanins, mucilages, tannins, and phenolic compounds. Both extracts exhibited antibacterial activity, with LsOE and the natural mouthwash showing intermediate efficacy (MIC 128 µg/mL), while PaEE alone was less effective (MIC 512 µg/mL). The formulated mouthwash (F1) significantly reduced bacterial growth, and the combined use of LsOE and PaEE indicated a synergistic effect. These findings support the potential of plant‐based formulations for dental biofilm control and encourage further research to expand their applicability in public oral health.

## Graphical abstract

Plant‐Based Antibacterial Mouthwash.

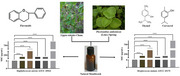

## 1 Introduction

Dental caries and periodontal disease are oral conditions that can be controlled through effective plaque management, and several plants are described in the literature as allies in this control and in other oral conditions [1]. When neglected, oral diseases can significantly affect quality of life, compromising basic functions such as chewing, speech, and social interaction, in addition to worsening preexisting systemic conditions, highlighting the importance of a preventive and integrated approach in public health [2].

The use of medicinal plants represents a viable and promising alternative due to the wide availability of natural resources and the knowledge accumulated by ethnomedicine over generations [3]. The chemical composition of medicinal plants may offer a unique combination of antimicrobial, antioxidant, and anti‐inflammatory properties [4], making them excellent candidates for applications in oral health. Thus, the development of natural mouthwashes leverages these benefits while addressing the growing public demand for healthier products, free of synthetic compounds and with reduced environmental impact [5].

*Plectranthus amboinicus* (Lour.) Spreng. has attracted considerable interest in the field of oral health due to its antibacterial properties, which represent a viable and sustainable alternative for managing dental biofilms and preventing caries and periodontal diseases [6]. Silva et al. [7] demonstrated the efficacy of the ethanolic extract of *P. amboinicus*, rich in flavonoids and phenolic acids, against Gram‐positive bacteria such as *Staphylococcus aureus* strains isolated from patients with otitis externa.

*Lippia sidoides* Cham. exhibits antimicrobial properties mainly attributed to the high levels of thymol and carvacrol found in its leaf essential oil [8]. These compounds not only inhibit bacterial and fungal growth but also demonstrate synergistic action when combined with other antimicrobial agents, disrupting the cellular membrane integrity of resistant microorganisms, including *Streptococcus mutans* and *S. aureus* [9].

Studies indicate that synergy among bioactive compounds from different plant species can enhance the therapeutic efficacy of formulations. For instance, the combination of *P. amboinicus* extract with *L. sidoides* essential oil may potentiate antimicrobial effects against resistant bacteria, while providing a broader spectrum of action against fungi and other oral pathogens [10].

Although both extracts have been individually studied in dental formulations, there is still a scarcity of research investigating the combination of *P. amboinicus* and *L. sidoides* in a single product with synergistic potential. This study aims to address this gap by evaluating their combined activity against cariogenic and pathogenic strains relevant to dentistry. From this perspective, the present study proposed to assess the antibacterial capacity of *P. amboinicus* and *L. sidoides* together against *S. mutans* and *S. aureus* in order to support the development of a mouthwash capable of disrupting dental biofilm and contributing to caries prevention.

## 2 Results and Discussion

### 2.1 Chemical Characterization of the Ethanolic Extract of *Plectranthus amboinicus*

The PaEE extract showed the presence of flavonoids (flavones, flavanones, xanthones, chalcones, and aurones), triterpenoids, tannins, and saponins, as shown in Table 1. The species *P. amboinicus* is known to possess a chemical composition rich in flavonoids such as apigenin, chrysoeriol, cirsimaritin, luteolin, quercetin, salvigenin, and taxifolin. In addition, reducing sugars, triterpenic acids, tannins, amino groups, and triterpenic steroids have also been detected in its leaves, alongside the absence of alkaloids [11], findings that are corroborated by the present study.

**TABLE 1:** Classes of secondary metabolites identified in the ethanolic extract of *Plectranthus amboinicus*.

Extract	Secondary metabolites													
	CT	PT	TT	LA	AC	F	FV	FVN	XT	CH	AR	CQ	AL	SP
PaEE	+	+	+	—	+	+	—	+	+	—	+	—	—	+

Abbreviations: AC, anthocyanins and anthocyanidins; AL, alkaloids; AR, aurones; CH, chalcones; CQ, catechins; CT, condensed tannins; F, flavones; FV, flavonols; FVN, flavanones; LA, leucoanthocyanidins; PT, pyrogallol tannins; SP, saponins; TT, triterpenoids; XT, xanthones; (+), present; (–), absent.

PaEE exhibited total phenolic and flavonoid contents of 40 mg GAE/g and 40.58 mg QE/g, respectively. Faisal et al. [12], studying plantlet regeneration of *P. amboinicus*, reported phenolic and flavonoid contents of 56.64 mg GAE/g and 40.27 mg QE/g, respectively, corroborating the findings obtained here. In contrast, Ślusarczyk et al. [13], evaluating the chemical profile of different cultivars from Indonesia and Poland, found phenolic levels ranging from 8.89 to 112.95 mg GAE/g and flavonoid levels between 3.77 and 96.83 mg QE/g. This variability within the same species may be related to genetic factors, age, seasonal variation, cultivation practices, and storage conditions to which samples were subjected [14].

### 2.2 Chemical Characterization of the Essential Oil of *Lippia sidoides*

**FIGURE 1:**
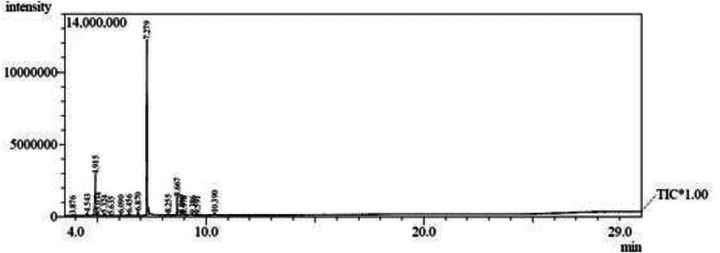
GC/MS chromatogram of the essential oil from *Lippia sidoides* leaves.

Among the 17 compounds identified in LsEO (Figure 1), thymol stands out as the major constituent, accounting for 59.25%, followed by cymene (20.81%) and caryophyllene (7.23%), as shown in Table 2.

**TABLE 2:** GC/MS chemical composition of the essential oil from *Lippia sidoides* leaves.

No.	Compound	%	RT
1	α‐Phellandrene	1.15	3.876
2	β‐Myrcene	1.42	4.543
3	Cymene	20.81	4.915
4	Eucalyptol	2.16	5.034
5	δ‐Terpinene	0.95	5.324
6	Ocimene	0.48	5.635
7	Myrtenol	0.69	6.090
8	4‐Terpineol	0.82	6.456
9	Methylthymyl ether	1.43	6.870
10	Thymol	59.25	7.279
11	Copaene	0.60	8.255
12	Caryophyllene	7.23	8.667
13	Aromadendrene	0.65	8.860
14	α‐Humulene	0.37	8.978
15	Ledene	0.59	9.386
16	Δ‐Cadinene	0.41	9.591
17	Caryophyllene oxide	0.99	10.390
**Total**	—	**100**	—

Abbreviation: RT, retention time.

These compounds are well known for their biological activities, including antimicrobial and antioxidant effects, which confer significant therapeutic potential to the oil [15]. The identification and quantification of essential oil components are fundamental for validating their efficacy and their application in health‐related products, such as mouthwashes or other natural treatments.

Li et al. [16] demonstrated that thymol disrupts cellular homeostasis and inhibits the growth of *S. aureus* by damaging the cell membrane, decreasing NADPH concentration, increasing NADP^+^ levels, and promoting lipid peroxidation. Almeida‐Bezerra et al. [17] showed that caryophyllene exhibits synergistic activity when combined with ampicillin, reducing the MIC of this antibiotic by 50% against the QacA efflux pump of *Staphylococcus aureus* K4414 in vitro, matching the performance of the standard efflux pump inhibitor chlorpromazine.

### 2.3 Minimum Inhibitory Concentration (MIC)

The strains were sensitive to all tested samples, as shown in Table 3. The PaEE and   LsEO mixture exhibited the highest inhibitory efficacy against bacterial growth, with an MIC of 64 µg/mL against *S. mutans* ATCC 25175, indicating a synergistic effect, since their isolated MICs were 512 and 128 µg/mL, respectively. The natural mouthwash showed an MIC of 128 µg/mL against both bacteria, demonstrating lower efficacy than the commercial control.

**TABLE 3:** Minimum inhibitory concentration (MIC).

Samples	MIC (µg/mL)	
	*S. mutans* ATCC 25175	*S. aureus* ATCC 25923
Natural mouthwash	128 ± 0.0	128 ± 0.0
Commercial control	8 ± 0.0	8 ± 0.0
LsOE + PaEE	64 ± 0.0	128 ± 0.0
LsOE	128 ± 0.0	128 ± 0.0
PaEE	512 ± 0.0	512 ± 0.0

Abbreviations: LsEO, *Lippia sidoides* essential oil; PaEE, *Plectranthus amboinicus* ethanolic extract.

**FIGURE 2:**
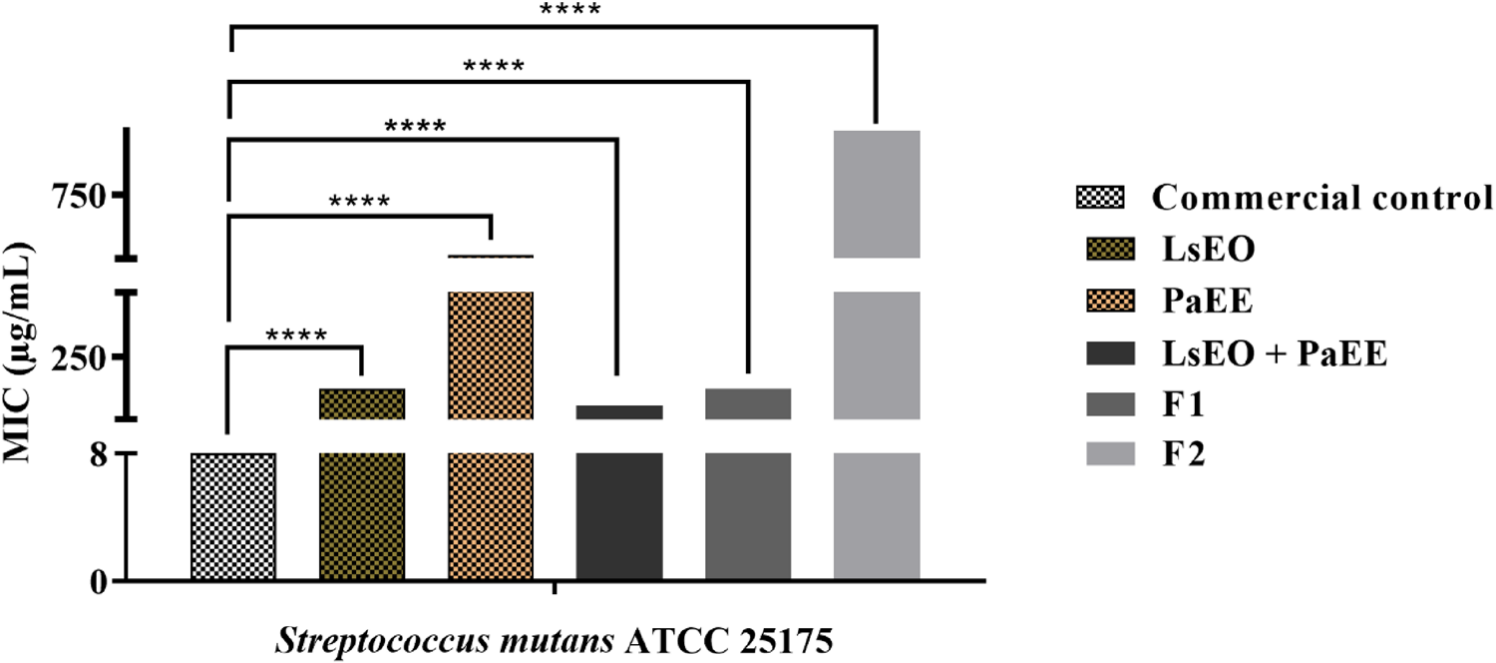
Antibacterial effect against *Streptococcus mutans* ATCC 25175. Two‐way ANOVA followed by Bonferroni post‐test. ****p < 0.0001. F1, natural mouthwash formulation; F2, negative mouthwash control; LsEO, *Lippia sidoides* essential oil; PaEE, *Plectranthus amboinicus* ethanolic extract.

Based on the results obtained for *S. mutans* (Figure 2), which differed statistically from the negative control, the formulations containing natural compounds exhibited relevant antibacterial activity, reinforcing their potential as viable alternatives. The combination of extracts, represented by formulation F1, confirms the importance of synergy between the bioactive compounds present in the plants used. This effectiveness highlights the robustness of the natural formulation in achieving levels close to those of widely used synthetic dental agents.

According to the literature, the flavonoids and tannins present in *P. amboinicus* extract act as antibacterial agents by interfering with bacterial energy metabolism and contributing to the inhibition of *S. mutans* growth [18, 19]. The synergy between these compounds is likely responsible for the promising microbiological results observed [6]. In contrast, previous studies indicate that thymol, present in rosemary essential oil, has potent antimicrobial properties due to its ability to destabilize bacterial membranes and inhibit the synthesis of essential proteins [8].

**FIGURE 3:**
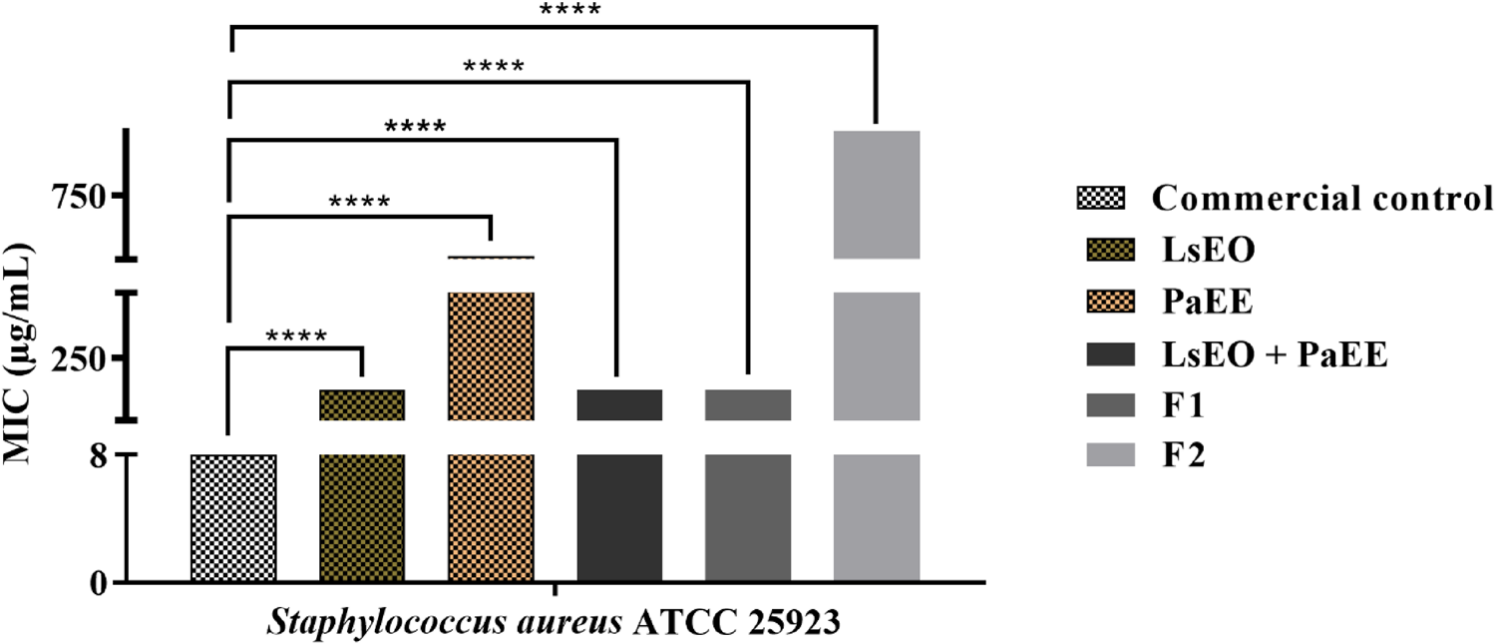
Antibacterial effect against *Staphylococcus aureus* ATCC 25923. Two‐way ANOVA followed by Bonferroni post‐test. ****p < 0.0001. F1, natural mouthwash formulation; F2, negative mouthwash control; LsEO, *Lippia sidoides* essential oil; PaEE, *Plectranthus amboinicus* ethanolic extract.

The results obtained for *S. aureus* (Figure 3) also differed statistically from the negative control and followed the same pattern of reduction observed for the previous strain. The MIC values for LsEO, PaEE, and especially formulation F1 are particularly important given that this strain is known for forming resistant biofilms and presenting increasing antibiotic resistance, such as methicillin‐resistant strains (MRSA) [20].

The observed synergy may be related to interactions between the water‐soluble phenolics of *P. amboinicus* and the lipophilic monoterpenes of *L. sidoides*, promoting multiple mechanisms of bacterial inhibition, such as membrane disruption, metal ion chelation, and interference with quorum‐sensing communication [21, 22].

*S. aureus* is particularly challenging due to its ability to form biofilms on abiotic and biotic surfaces, such as dental prostheses and implants, providing a protective barrier that hinders antimicrobial penetration and favors infection persistence [23]. Studies indicate that the phenolic and terpenoid compounds present in the extracts used exhibit anti‐adhesive and anti‐quorum‐sensing activities, which can be applied in dental formulations to prevent infections associated with dental devices [24]. Kim et al. [25] demonstrated that thymol reduces *S. aureus* biofilm formation by decreasing cell surface hydrophobicity, as well as reducing the strain's resistance to ultraviolet radiation and hypochlorous acid.

Although chlorhexidine is widely effective, its prolonged use is associated with undesirable side effects, such as dental staining, taste alteration, and potential impact on the beneficial oral microbiota [6]. Natural formulations, on the other hand, do not exhibit these adverse effects and offer additional advantages, such as anti‐inflammatory and antioxidant properties, contributing to broader oral health benefits. This reinforces the feasibility of using natural extracts as therapeutic alternatives, especially for long‐term applications [26].

## 3 Conclusions

The tests demonstrated that the bioactive compounds present in *Plectranthus amboinicus* and *Lippia sidoides* exhibit antibacterial activity, validating their ability to disrupt dental biofilms and prevent the development of caries and other oral infections. This reinforces the potential of these plants as effective alternatives in combating microorganisms associated with oral diseases, such as *Streptococcus mutans* and *Staphylococcus aureus*.

The results presented broaden the prospects for new research and innovations in the field of dentistry, highlighting the preventive potential of the developed formulations. Future studies may focus on in vivo clinical trials to assess the product's effectiveness under real conditions, as well as on combinations with other therapeutic approaches, such as the use of probiotics, thereby consolidating its position as a sustainable and scientifically supported alternative.

## 4 Experimental Section

### 4.1 Plant Material and Extract Preparation

The leaves of *Plectranthus amboinicus* were collected in the municipality of Juazeiro do Norte, Ceará, Brazil (07°12'47'' S; 39°18'55'' W). A voucher specimen was deposited in the Dárdano de Andrade Lima Herbarium of the Regional University of Cariri under registration number 3037. A total of 1000 g of fresh *P. amboinicus* leaves were macerated in 99% ethanol for 72 h, and the resulting solution was filtered and evaporated under reduced pressure at 50°C using a rotary evaporator, yielding the ethanolic extract (PaEE) with a 1.30% (m/m) yield. The essential oil from *Lippia sidoides* leaves (LsEO) was provided by the Laboratory of Natural Products Research of the Regional University of Cariri (LPPN/URCA).

### 4.2 Chemicals and Solvents

All chemicals were of analytical grade. Ethanol (EtOH) and dichloromethane were purchased from Merck KGaA (Darmstadt, Hesse, Germany). Folin–Ciocalteu reagent, Na_2_CO_3_, AlCl_3_, quercetin, gallic acid, Brain Heart Infusion Broth, dimethyl sulfoxide, resazurin, saccharin, sodium benzoate, glycerin, and NaOH were obtained from Sigma‐Aldrich (St. Louis, Missouri, USA).

### 4.3 Identification of Secondary Metabolite Classes

Chemical prospecting was carried out following the methodology described by Matos [27] and Simões et al. [28] to identify the classes of secondary metabolites present in PaEE. The test is qualitative and consists of visual observation of color change or precipitate formation after the addition of specific reagents to the sample solutions.

### 4.4 GC/MS Analysis of *Lippia sidoides* Essential Oil

The sample was analyzed using a Shimadzu GC‐MS QP2010 system (Shimadzu Scientific Instruments Inc., Columbia, MD, USA) equipped with a fused‐silica capillary column SH‐Rtx‐5 (30 m × 0.25 mm i.d.; 0.25 µm film thickness). The temperature program was as follows: 80°C–180°C at 4°C/min, then 180°C–246°C at 6.6°C/min, followed by 10 min at 280°C at 3.4°C/min, totaling 30 min of analysis. Helium was used as the carrier gas at 1.5 mL/min in split mode (1:100), and the injection port was set to 220°C. Quadrupole MS parameters were as follows: interface temperature 280°C; ion source temperature 200°C; electron ionization at 70 eV; mass scanning range 40–350 m/z at 1.0 scan/s. The injection volume was 1 µL of a 500‐ppm solution prepared in 99.9% UV/HPLC‐grade dichloromethane. Constituents were identified by comparison with mass spectral databases (NIST 08) and with authentic spectra reported in the literature [29].

### 4.5 Total Phenols

The total phenolic content of PaEE was determined using the Folin–Ciocalteu method, following Nonato et al. [30], with modifications. The reaction medium consisted of 25 µL of extract (50–1000 µg/mL in ethanol), 625 µL of freshly prepared 10% Folin–Ciocalteu reagent diluted in water, and 500 µL of 7.5% Na_2_CO_3_. The mixture was protected from light, incubated for 15 min at 45°C in a water bath, and the absorbance was measured at 735 nm. The assay was performed in triplicate, and results were expressed as milligrams of gallic acid equivalent per gram of extract (mg GAE/g).

### 4.6 Total Flavonoids

The total flavonoid content was determined using the aluminum chloride colorimetric method according to Zhishen, Mengcheng, and Jianming [31], with adaptations. Sample aliquots (1.5, 2.5, and 3.5 µg/mL) were mixed with 2.0 mL ethanol and 1 mL of 15% AlCl_3_. Samples were incubated at room temperature for 30 min, and absorbance was measured at 425 nm. Experiments were performed in triplicate, and quercetin was used as the calibration standard (mg QE/g).

### 4.7 Antibacterial Activity

The antibacterial assays were performed using *Streptococcus mutans* INCQS 00446 and *Staphylococcus aureus* ATCC 25923 strains supplied by Fundação Oswaldo Cruz (FIOCRUZ), Brazil. MIC was evaluated using the broth microdilution method [32]. The inoculum was activated in Brain Heart Infusion Broth (BHI, 3.8%) for 24 h at 35°C ± 2°C, standardized to 1 × 10^8^ CFU/mL (0.5 McFarland). The suspension was diluted to 1 × 10^6^ CFU/mL in 10% BHI broth, and 100 µL were added to microdilution plate wells containing extract concentrations. The final inoculum was 5 × 10^5^ CFU/mL. Extracts were diluted in DMSO and distilled water to obtain a final solution of 1024 µg/mL. Serial dilutions ranging from 512 to 8 µg/mL were prepared. Plates were incubated for 24 h at 35°C ± 2°C, and readings were taken by colorimetry using 25 µL of 0.01% resazurin.

### 4.8 Mouthwash Formulation

The PaEE and LsEO solutions were prepared according to the MIC results. The mouthwash formulation followed the methodology adapted from Zanin et al. [33]. Components and respective quantities used for preparing 20 mL of the natural mouthwash are shown in Table 4.

**TABLE 4:** Components used for formulating 20 mL of natural mouthwash.

Component	F1	F2
PaEE (512 µg/mL)	0.02 g	—
LsEO (128 µg/mL)	0.02 g	—
Saccharin	0.03 g	0.03 g
Sodium benzoate	0.06 g	0.06 g
Glycerin	0.8 mL	0.8 mL
Distilled water	20 mL	20 mL
Mint essence	0.08 mL	0.08 mL
Sodium hydroxide 10%	qs pH 6.3	qs pH 6.3

The preparation consisted of cold mixing of all raw materials under stirring until complete incorporation. Formulation 1 (F1) represents the natural mouthwash, whereas formulation 2 (F2) corresponds to the negative control. Commercial chlorhexidine gluconate mouthwash (0.12%) was used as the positive control.

This study was conducted in compliance with good laboratory practice guidelines and did not involve human or animal subjects, therefore not requiring ethics committee approval.

### 4.9 Statistical Analysis

The antibacterial assay results were expressed as mean ± standard deviation from up to eight replicates (*n* = 8). Data were analyzed using two‐way ANOVA followed by Bonferroni post‐test, and results with *p* < 0.05 were considered statistically significant. Statistical analyses were performed using GraphPad Prism 9.3 (GraphPad Software, San Diego, California, USA).

## Author Contributions

**Wilma Francisca da Silva**: conceptualization, investigation, formal analysis, writing – original draft. **Carla de Fatima Alves Nonato**: investigation. **Débora Odília Duarte Leite**: investigation. **Fabiola Fernandes Galvão Rodrigues**: formal analysis, writing – review and editing. **Fazia Fernandes Galvão Rodrigues**: investigation. **Ana Cecília Calixto Donelardy**: investigation. **José Galberto Martins da Costa**: conceptualization, writing – review and editing, supervision.

## Conflicts of Interest

The authors declare no conflicts of interest.

## Data Availability

The data that support the findings of this study are available from the corresponding author upon reasonable request.
